# Conservative Surgical Management of a Pulmonary Hydatid Cyst in an Adolescent Having Extra-pulmonary Lesions by a Multi-disciplinary Approach

**DOI:** 10.7759/cureus.58600

**Published:** 2024-04-19

**Authors:** Vishal V Bhende, Jignesh B Rathod, Ashwin S Sharma, Jigar P Thacker, Mathangi Krishnakumar, Saptak P Mankad, Deepakkumar V Mehta, Hemlata V Kamat, Birva N Khara, Sanket H Mehta, Dhavalkumar Prajapati, Amit Kumar, Mansi Chaudhary, Kuldeep V Kotadiya, Aradhanaba B Gohil, Prachi P Vani, Sweta R Panchal, Nili J Mehta, Divyanshi A Patel, Vidit A Gadoya, Himanshu D Ghoti

**Affiliations:** 1 Pediatric Cardiac Surgery, Bhanubhai and Madhuben Patel Cardiac Centre, Shree Krishna Hospital, Bhaikaka University, Karamsad, IND; 2 Surgery, Pramukhswami Medical College, Shree Krishna Hospital, Bhaikaka University, Karamsad, IND; 3 Internal Medicine, Gujarat Cancer Society Medical College, Hospital, and Research Centre, Ahmedabad, IND; 4 Pediatrics, Pramukhswami Medical College, Shree Krishna Hospital, Bhaikaka University, Karamsad, IND; 5 Anesthesiology, St. John's Medical College Hospital, Bengaluru, IND; 6 Internal Medicine, Dev Medical Hospital, Vadodara, IND; 7 Radiodiagnosis and Imaging, Pramukhswami Medical College, Shree Krishna Hospital, Bhaikaka University, Karamsad, IND; 8 Anesthesiology, Pramukhswami Medical College, Shree Krishna Hospital, Bhaikaka University, Karamsad, IND; 9 Pulmonary Medicine, Pramukhswami Medical College, Shree Krishna Hospital, Bhaikaka University, Karamsad, IND; 10 Pediatric Intensive Care Unit (PICU), Bhanubhai and Madhuben Patel Cardiac Centre, Shree Krishna Hospital, Bhaikaka University, Karamsad, IND

**Keywords:** lobectomy, pulmonary hydatid disease, hydatid cyst of liver, capitonnage, cystotomy, cystectomy

## Abstract

*Echinococcus granulosus* causes hydatid cysts, a significant zoonotic and pulmonary parasitic disease that can mimic various pathologies and is often harder to manage than the disease itself. A hydatid cyst is considered a significant health problem in India, Iran, China, and Mediterranean countries, which lack satisfactory environmental health, preventive medicine, and veterinarian services. Echinococcosis continues to be a major community health burden in several countries, and in some terrains, it constitutes an emerging and re-emerging disease. Cystic echinococcosis is the most common human disease of this genus, and it accounts for a significant number of cases worldwide. Herein, a case involving an 11-year-old presenting with fever, dry cough, and right hypochondrial pain is presented, where imaging revealed a hydatid cyst in the lung. Surgical removal of the cyst was achieved through right posterolateral thoracotomy under one-lung ventilation and anesthesia using intubation with a double-lumen endotracheal tube (DLET or DLT), highlighting surgery as the primary treatment despite the lack of consensus on surgical methods. This case underscores the effectiveness of individualized, parenchyma-preserving surgery for even large, uncomplicated cysts, indicating a positive prognosis.

## Introduction

The parasite Echinococcus species, Cestode (small tapeworms of carnivorous animals), causes a zoonosis known as a hydatid cyst. Two predominant species affect humans, namely, *Echinococcus granulosus* and *Echinococcus multilocularis*. The hydatid cyst was first described by Hippocrates as a “liver full of water” [1]. Humans are accidental hosts of cystic echinococcosis, typically spread by infected household animals like dogs and livestock [2].

Generally, the lesion is a thick jelly-like endocyst filled with several daughter cysts and larvae. The healthy lung parenchyma is lined with a thick fibrotic layer covering the endocyst, possibly due to an inflammatory response from the host. Hydatid cysts can occur anywhere, like the brain, liver, lungs, kidney, spleen, and soft tissue, often attacking the liver and lungs. Pulmonary disease is more common in young people as they are more exposed to infected animals [3]. The lung’s elastic structure allows hydatid cysts to grow and invade it faster than in the liver [4]. Furthermore, negative intrathoracic pressures may promote quick growth of the pulmonary cyst [5]. The rupture rate of pulmonary hydatid cysts is higher than that of hepatic hydatid cysts [6]. Most cases of pulmonary hydatid cysts in children are discovered incidentally or as a result of respiratory symptoms such as coughing, shortness of breath, chest discomfort, hemoptysis, and fever.

Pulmonary hydatid cysts are typically treated with surgery, with pulmonary cystectomy and capitonnage serving as the main preference since they preserve lung tissue. However, when there are complications, such as severe bleeding, bronchiectasis, chronic abscess, or serious parenchymal damage, lobectomy or even pneumonectomy is advised [7, 8].

## Case presentation

**Figure 1:**
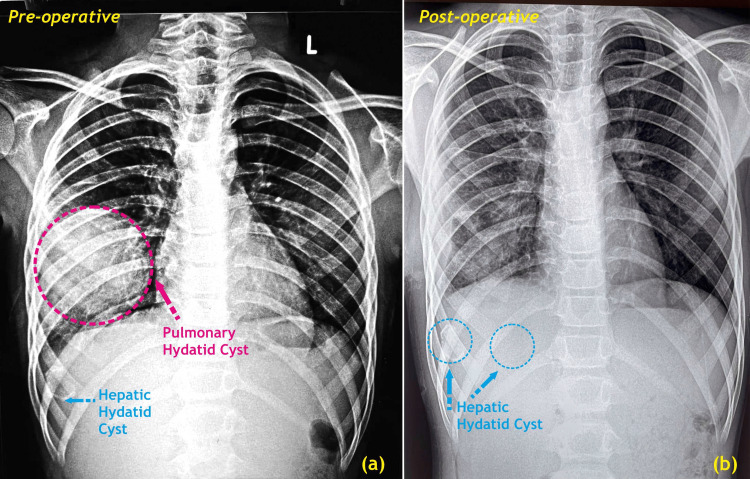
(a) Pre-operative chest X-ray revealing dense homogeneous radio-opaque opacity in the right lower lobe of the lung (red marking) with calcified hepatic hydatid cysts (blue marking); (b) Post-operative chest X-ray without opacity but with persisting hepatic hydatid cysts (blue marking) Image credits: Dr. Vishal V. Bhende

An 11-year-old male adolescent reported to the pediatric clinic of our hospital complaining of a dry cough and moderate-grade fever with abdominal pain but without dyspnea, chest pain, or weight loss. A detailed history-taking revealed that the patient had multiple visits to hospitals over the past years with complaints of fever and cough. The patient was managed with supportive therapy in the form of antipyretics and antitussives. The patient had relief with these for a few months, followed by a recurrence of symptoms. The fever was low-moderate, with no chills or rigor and diurnal variations. The cough was episodic and not associated with sputum production, hemoptysis, dyspnea, or chest pain. There were no associated joint pains or rashes. A chest X-ray confirmed the diagnosis of a pulmonary hydatid cyst (Figure 1).

**Figure 2:**
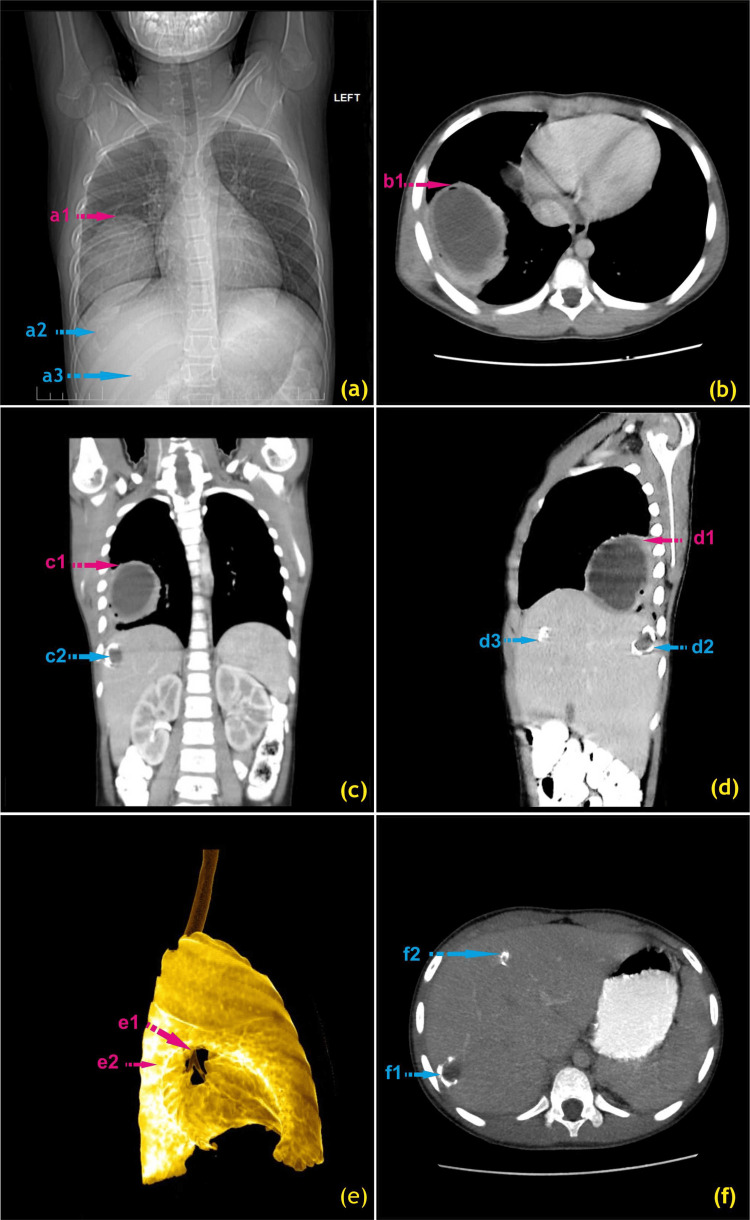
(a) A pre-operative CT scanogram shows big lung opacity, big liver calcification (a2), and small liver calcification (a3). (b) A contrast CT (axial image) shows a double-layered hydatid cyst in the right lower lobe (RLL) with air between layers (b1). (c) A contrast CT (coronal reformation) shows a double-layered lung hydatid cyst in RLL with air between layers (c1) and a calcified liver hydatid cyst in Couinaud segment VII (c2). (d) An oblique sagittal image shows a double-layered lung hydatid cyst with air between layers (d1), a calcified liver hydatid cyst in segment VII (d2), and a small calcified liver cyst in segment IVA (d3). (e) A pseudo-colored minimum intensity projection lateral image of the right lung shows that the lung was destroyed by a lung hydatid cyst (e1) and that the bronchi are intact (e2). (f) Two liver hydatid cysts (axial image) are seen where there is a big calcified hydatid cyst in segment VII (f1) and a small calcified hydatid cyst in segment IVA (f2). Pulmonary hydatid cyst: red marking; hepatic hydatid cyst: blue marking Image credits: Dr. Deepakkumar V. Mehta

A CT of the chest and upper abdomen was also done (Figure 2).

The chest CT scan revealed a big, well-defined, oval cystic lesion in the lateral basal segment of the right lower lobe (RLL), extending to the adjacent lateral portions of the superior, anterior, basal, and posterior basal segments of the RLL. The cyst had a well-defined, mildly enhancing, peripheral, thick wall with a thin, mildly enhancing wall just internal to it, as well as a few very small, peripheral air foci lying in the lesion, mainly in its wall. The cyst had maximum dimensions of 7.52 cm × 6.25 cm × 6.65 cm, associated with small areas of collapse postero-supero-laterally, posteriorly, postero-inferiorly, and postero-infero-laterally.

In addition to the pulmonary hydatid cyst, there appeared to be a non-enhancing hypodense cystic lesion with incomplete wall calcification in segment VII of the right lobe of the liver, measuring approximately 1.9 × 1.8 cm. Another soft tissue lesion with near-complete calcification was observed in segment IV of the left lobe of the liver, measuring approximately 0.7 × 0.6 cm.

Anesthesia and pain relief

The patient was referred for surgery with the American Society for Anesthesiologists (ASA) III consent. The surgery required one lung ventilation; hence, a double-lumen endotracheal tube (DLET) No. 26 Fr. was used. The pre-anesthesia protocol included nebulization with normal saline. Dexmedetomidine injection (inj) was started at a loading dose of 1 mcg/kg over 20 minutes and then continued at the maintenance dose of 0.5 mcg/kg/hr; IV midazolam 0.5 mg. was given just before transfer to the operating room. General anesthesia was administered via an injection of fentanyl, propofol, and succinylcholine using a DLET No. 26 Fr. Pain relief was achieved by an epidural infusion of inj. bupivacaine (0.25%) and inj. fentanyl (1 mcg/cc), with a total dose of 10 ml administered via a thoracic epidural catheter after induction of anesthesia. Anesthesia was maintained using an inhalation agent, sevoflurane, an IV dexmedetomidine infusion, inj. vecuronium, and a 50% fraction of inspired oxygen (FiO2), with controlled ventilation. Towards the end of the surgery, blood gas analysis results were normal, and the patient was successfully extubated on the operation table. Post-operatively, analgesia was achieved with a top-up dose of inj. bupivacaine 0.0625 mg/cc + inj. fentanyl 0.25 mcg/cc via the epidural route and IV paracetamol.

Operative technique

**Figure 3:**
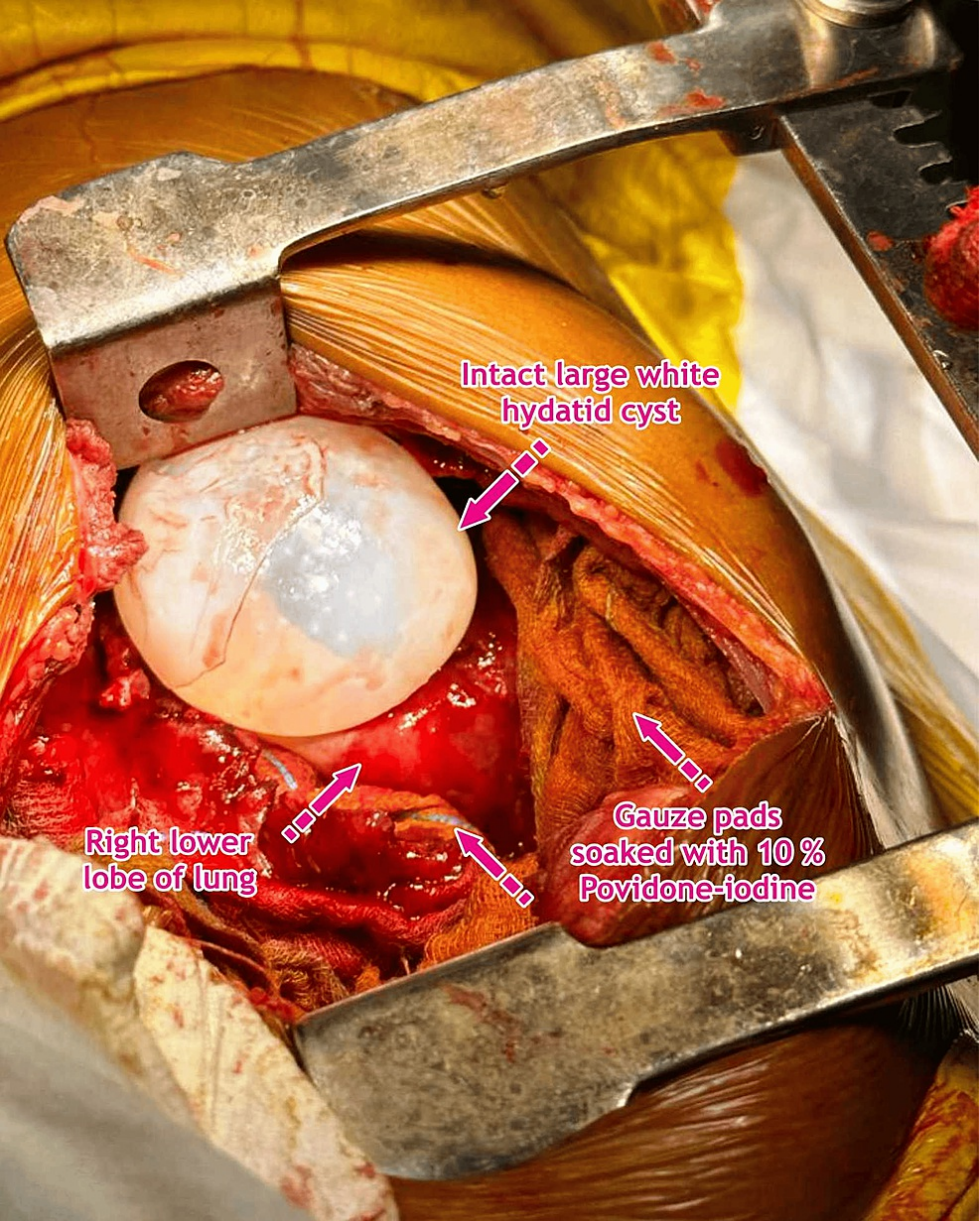
A very large white cyst is seen during the right posterolateral thoracotomy of the right lung, which is surrounded by gauze/sponges soaked with 10% povidone-iodine (Betadine solution). Image credits: Dr. Vishal V. Bhende

**Figure 4:**
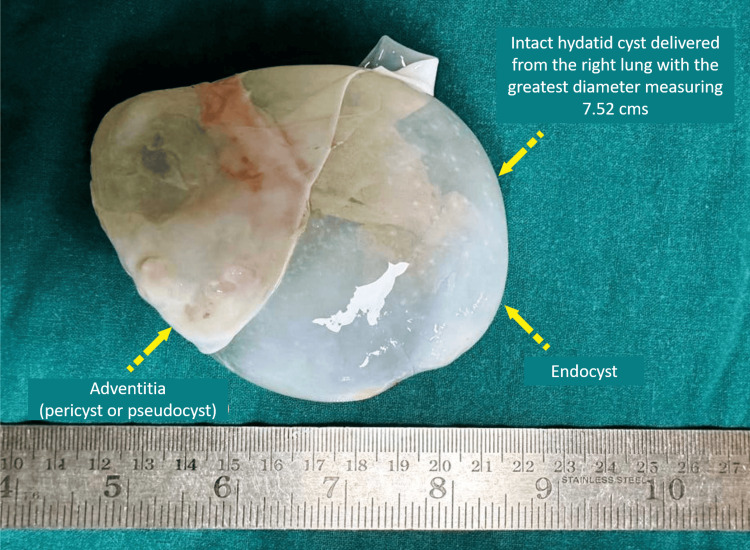
Hydatid cyst specimen delivered in-toto after surgical excision (enucleation) Image credits: Dr. Vishal V. Bhende

The surgical approach applied was right posterolateral thoracotomy as per the location of the pulmonary hydatid cyst, accessed via the right fifth intercostal space. The preferred surgical procedure was cystectomy with capitonnage. After locating the pulmonary hydatid cyst, the surgical incision and surrounding tissue were covered with packed gauze soaked in 10% povidone-iodine, exposing only the cyst-containing lung area (Figures 3-4, Video 1).

**Video 1:** Surgery of the hydatid cyst: Barrett’s method in 3D medical animation Video credits: Dr. Vishal V. Bhende

Cystectomy (using Barrett’s method) and closure of the bronchial openings were performed with 10% povidone-iodine irrigation while the anesthesiologist inflated the lung. The cyst space was obliterated via capitonnage after closing the bronchial openings and the surrounding tissues with 2/0 Vicryl sutures (according to the incision size) without pledgets.

**Figure 5:**
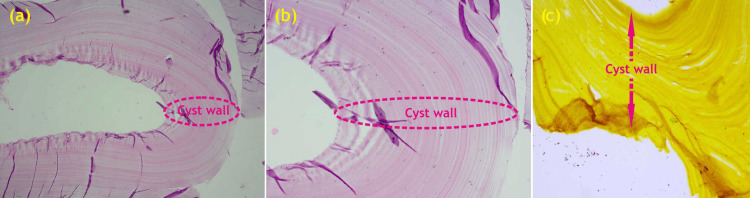
(a) H&E (4x2): The microphotograph shows the presence of the outer acellular laminated membrane (H&E stain, 40x); (b) H&E (10x1): The microphotograph shows the focal presence of a transparent nucleated lining beneath the laminated membrane (H&E stain, 100x); (c) Grocott methenamine silver (GMS) stain H&E: hematoxylin & eosin Image credits: Dr. Mustafa Ranapurwala

The specimen delivered was sent for histopathological examination, which provided definitive evidence of echinococcosis showing acellular laminated membrane, a hallmark of hydatidosis. However, it did not reveal signs of secondary infection, cyst rupture, or infiltration into surrounding tissues, which are critical for assessing the patient's prognosis and potential complications. Formalin-fixed paraffin-embedded sections of the cyst wall were stained with hematoxylin and eosin stain and performed sequentially with Grocott methenamine silver (GMS) stain (Figure 5).

The patient had an uneventful recovery with no complications. Albendazole was administered at 10 mg/kg/day (400 mg twice daily) due to extra-pulmonary involvement (hepatic cysts). After 28 days, medications were stopped for seven days. Follow-up liver function tests were conducted once a month for the first three months, which were then continued every three months until the end of the first post-operative year. Table 1 summarizes the patient demographics, clinical characteristics, and cyst findings.

**Table 1:** Patient demographics, clinical characteristics, and cyst findings

Parameters	Description
Age	11 years
Sex	Male
Height	135 cm
Weight	22 kg
Cyst localization	Right lower lung lobe
Liver involvement	Positive
Cyst dimensions	<10 cm
Complications	Negative
Length of hospital stay	10 days

## Discussion

Cystic echinococcosis (CE) is the most common human disease of this genus, and it accounts for >95% of the estimated two to three million cases worldwide [9].

**Figure 6:**
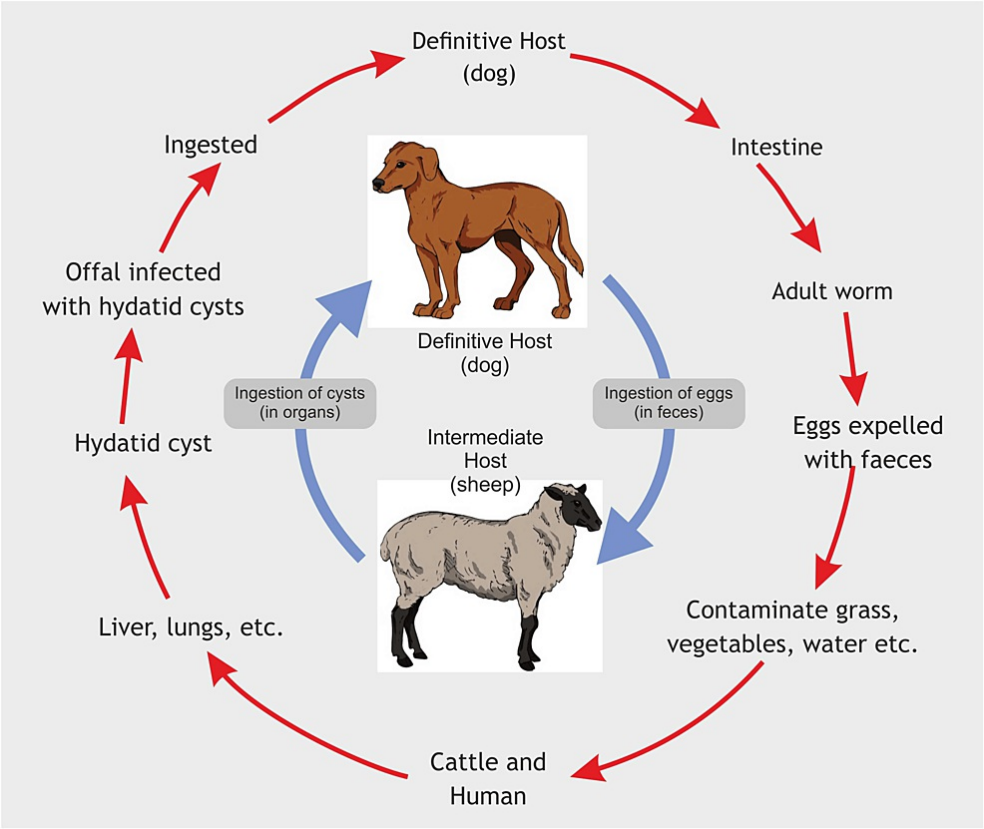
Life cycle of Echinococcus granulosus Image credits: Dr. Vishal V. Bhende

Hydatid cysts remain a public health problem, particularly in echinococcosis-endemic developing countries. Children account for approximately 10%-20% of hydatid cyst cases [6]. Pulmonary hydatid cysts usually develop when larvae enter the circulation and spread through the hepatic sinusoids and, less frequently, the lymphatic circulation (Figure 6) [6]. In children, 64% of hydatid cysts occur in the lung and 28% in the liver, unlike adults [10]. Pulmonary hydatid cysts are more symptomatic due to the compressibility, higher vascularity, and lower negative pressure of the lung tissue [11]. Pulmonary hydatid cysts are capable of growing more than 5 cm per year because of the high compliance and flexibility of the lungs [12].

Previous studies have demonstrated that men are more likely to develop pulmonary hydatid cysts [8], as in our case, a male adolescent. Pulmonary hydatid cysts with a diameter of >10 cm are known as giant cysts. Pediatric series report a 15%-31% rate for giant pulmonary cysts [13]. Some authors suggest that the rupture rate increases with increased cyst diameter due to the thinner lung parenchyma surrounding it [14]. However, Kuzuan et al. observed no correlation between cyst diameter and rupture [15]. Contrarily, Burgos et al. indicated that cyst enlargement was a negative risk factor for rupture [16]. Akgul Ozmen et al. found that the unruptured group had a significantly larger mean cyst diameter than the ruptured group [17]. In our study, cyst diameter did not increase or indicate signs of impending rupture.

Pulmonary hydatid cysts can infest all pulmonary lobes; however, they are more common in the RLL, as in our case [18]. Contrarily, Hamouri et al. reported a higher prevalence of ruptured and unruptured cysts in the left lower lobe [19].

Minimally invasive procedures, such as puncture-aspiration-injection-reaspiration (PAIR), can be used to treat liver hydatid cysts. The goal of surgical intervention includes the removal of the entire cyst while preserving the lung parenchyma as much as possible and without allowing intra-operative spillage. In our case, cystectomy with capitonnage (Barrett's method) was performed for the pulmonary hydatid cyst, and two small cystic lesions with wall calcification in segments VII and IVA of the liver were treated with albendazole and monitored on follow-up.

The patient had an uneventful recovery with on-table extubation and no complications. The various surgical options for pulmonary hydatid cysts are given in Table 2.

**Table 2:** Various surgical procedures for pulmonary hydatid disease Source: [20]

Surgical approach	Description
Enucleation (Ugon method)	In 1902, Dr. Ugon proposed enucleation, a procedure in which the hydatid cyst is surgically and completely excised from the lung parenchyma.
Pericystectomy (Perez-Fontana method)	In 1953, Perez Fontana proposed pericystectomy, which involves the excision of the hydatid cyst and the pericyst that adheres firmly to the normal lung parenchyma. In this procedure, the airway openings must be closed and the healthy lung parenchyma approximated.
Cystostomy with capitonnage (Barrett’s method)	Proposed by Barrett in 1952, this procedure involves cystostomy followed by capitonnage of the residual cystic cavity.
Capitonnage and bronchial tube closure following cystectomy (Posadas method)	Posadas, who was Barrett’s contemporary, modified Barrett’s procedure. In the modified procedure, the airway openings were closed before capitonnage.
Cystectomy with bronchial opening closure only	This method, recently attempted by Turna, Erdogan, and Eren et al., is similar to the Posadas technique but without capitonnage.
Figuera’s open aspiration technique	Figuera’s technique is comparable to the PAIR (puncture, aspiration, injection, and reaspiration) technique for hepatic hydatidosis treatment. Here, the cyst membranes and daughter cysts are suctioned.
Segmental resection	First performed by Liaras et al. in 1955, segmental resection involves the use of a technique similar to conventional resection for other disorders.
Lobectomy	Pulmonary lobectomy was first performed in 1950 for an inflammatory pulmonary lesion.
PAIR technique	It is performed after taking all necessary precautions/equipment, with CT/ultrasonography guidance and emergency drugs.

Preoperative albendazole treatment is a known risk factor for cyst wall rupture in pulmonary echinococcosis; thus, it is not recommended [21,22]. Albendazole is now routinely administered post-operatively for three or six months worldwide. We used this regimen in our case to monitor the liver hydatid cysts. The management of liver hydatid cysts often employs a conservative approach, particularly when lesions are asymptomatic, small, or in cases where surgical intervention poses a significant risk to the patient. This strategy is grounded in the understanding that small, uncomplicated cysts may remain stable for extended periods, and some may even regress [23].

## Conclusions

The diagnosis of hydatid cysts is suspected based on the presence of pulmonary cysts and a history of exposure to sheep and dogs in echinococcosis-endemic regions. Thus, pediatric patients with lower respiratory tract symptoms such as cough, hemoptysis, and fever should undergo further imaging, such as a thorax CT. Pulmonary hydatid cysts (PHCs) must be preferentially managed with parenchyma-sparing methods, which require sophisticated segmentectomy and lobectomy skills to avoid intra- and post-operative complications. All patients with hydatid cyst surgery should receive albendazole (10-15 mg/kg/day) taken twice daily for six months to prevent the recurrence of the disease. The risk of recurrence is as high as 11% if anti-helminths are not prescribed post-surgery. Albendazole is the drug of choice because of its higher bioavailability and the requirement of a minimum contact period of approximately 11 days. Continuous dosage administration has been found to be more efficacious than the earlier belief of an interrupted monthly dosage with a gap of two weeks to avoid hepatotoxicity. Patients with pulmonary hydatid cysts and a coexistent hepatic hydatid cyst, as in our case, do benefit from albendazole.

## References

[1] Pissiotis CA, Wander JV, Condon RE (1972). Surgical treatment of hydatid disease: prevention of complications and recurrences. Arch Surg.

[2] Qian ZX (1988). Thoracic hydatid cysts: a report of 842 cases treated over a thirty-year period. Ann Thorac Surg.

[3] Morar R, Feldman C (2003). Pulmonary echinococcosis. Eur Respir J.

[4] Halezeroglu S, Celik M, Uysal A, Senol C, Keles M, Arman B (1997). Giant hydatid cysts of the lung. J Thorac Cardiovasc Surg.

[5] Dincer SI, Demir A, Sayar A, Gunluoglu MZ, Kara HV, Gurses A (2006). Surgical treatment of pulmonary hydatid disease: a comparison of children and adults. J Pediatr Surg.

[6] Kabiri EH, El Hammoumi M, Kabiri M (2020). Surgical treatment of hydatidothorax in children: a retrospective study of 19 patients. J Pediatr Surg.

[7] Doğan R, Yüksel M, Cetin G (1989). Surgical treatment of hydatid cysts of the lung: report on 1055 patients. Thorax.

[8] Onal O, Demir OF (2017). Is anatomic lung resection necessary in surgical treatment of giant lung hydatid cysts in childhood?. Ann Thorac Cardiovasc Surg.

[9] Das R, Gupta V, Khullar S, Verma N, Mirdha BR (2023). Seropositivity pattern of human cystic echinococcosis at a tertiary care hospital of India. J Lab Physicians.

[10] Ksia A, Fredj MB, Zouaoui A (2020). Capitonnage seems better in childhood pulmonary hydatid cyst surgery. J Pediatr Surg.

[11] Cevik M, Boleken ME, Kurkcuoglu IC, Eser I, Dorterler ME (2014). Pulmonary hydatid disease is difficult recognized in children. Pediatr Surg Int.

[12] Aydogdu B, Sander S, Demirali O (2015). Treatment of spontaneous rupture of lung hydatid cysts into a bronchus in children. J Pediatr Surg.

[13] Sarkar M, Pathania R, Jhobta A, Thakur BR, Chopra R (2016). Cystic pulmonary hydatidosis. Lung India.

[14] Yuksel M, Kir A, Ercan S, Batirel HF, Baysungur V (1997). Correlation between sizes and intracystic pressures of hydatid cysts. Eur J Cardiothorac Surg.

[15] Kuzucu A, Ulutas H, Reha Celik M, Yekeler E (2014). Hydatid cysts of the lung: lesion size in relation to clinical presentation and therapeutic approach. Surg Today.

[16] Burgos L, Baquerizo A, Muñoz W, de Aretxabala X, Solar C, Fonseca L (1991). Experience in the surgical treatment of 331 patients with pulmonary hydatidosis. J Thorac Cardiovasc Surg.

[17] Akgul Ozmen C, Onat S (2017). Computed tomography (CT) findings of pulmonary hydatid cysts in children and the factors related to cyst rupture. Med Sci Monit.

[18] Kabiri EH, Kabiri M (2021). Clinical features and treatment of bronchial rupture of pulmonary hydatid cyst in children: a retrospective study of 36 patients. Gen Thorac Cardiovasc Surg.

[19] Hamouri S, Odat H, Syaj S, Hecker E, Alrabadi N (2021). Rupture of pulmonary hydatid cyst in pediatrics: a cross-sectional study. Ann Med Surg (Lond).

[20] Nabi MS, Waseem T (2010). Pulmonary hydatid disease: what is the optimal surgical strategy?. Int J Surg.

[21] Usluer O, Kaya SO, Samancilar O, Ceylan KC, Gursoy S (2014). The effect of preoperative albendazole treatment on the cuticular membranes of pulmonary hydatid cysts: should it be administered preoperatively?. Kardiochir Torakochirurgia Pol.

[22] Aydin Y, Ulas AB, Ince I (2022). Evaluation of albendazole efficiency and complications in patients with pulmonary hydatid cyst. Interact Cardiovasc Thorac Surg.

[23] WHO Informal Working Group (2003). International classification of ultrasound images in cystic echinococcosis for application in clinical and field epidemiological settings. Acta Trop.

